# Transplantation of mesenchymal stem cells genetically engineered to overexpress interleukin-10 promotes alternative inflammatory response in rat model of traumatic brain injury

**DOI:** 10.1186/s12974-018-1383-2

**Published:** 2019-01-05

**Authors:** S. T. Peruzzaro, M. M. M. Andrews, A. Al-Gharaibeh, O. Pupiec, M. Resk, D. Story, P. Maiti, J. Rossignol, G. L. Dunbar

**Affiliations:** 1 Field Neurosciences Institute of Laboratory for Restorative Neurology, Central Michigan University, Mt. Pleasant, MI 48859 USA; 2 Program in Neuroscience, Central Michigan University, Mt. Pleasant, MI 48859 USA; 3 Department of Psychology, Central Michigan University, Mt. Pleasant, MI 48859 USA; 4 Field Neurosciences Institute, St. Mary’s of Michigan, Saginaw, MI 48604 USA; 5 Department of Biology, Saginaw Valley State University, Saginaw, MI 48610 USA; 6 Brain Research Laboratory, Saginaw Valley State University, Saginaw, MI 48610 USA; 7 College of Medicine, Central Michigan University, Mt. Pleasant, MI 48859 USA

**Keywords:** Traumatic brain injury, Interleukin-10, Mesenchymal stem cells, Neuroinflammation, CD163

## Abstract

**Background:**

Traumatic brain injury (TBI) is a major cause for long-term disability, yet the treatments available that improve outcomes after TBI limited. Neuroinflammatory responses are key contributors to determining patient outcomes after TBI. Transplantation of mesenchymal stem cells (MSCs), which release trophic and pro-repair cytokines, represents an effective strategy to reduce inflammation after TBI. One such pro-repair cytokine is interleukin-10 (IL-10), which reduces pro-inflammatory markers and trigger alternative inflammatory markers, such as CD163. In this study, we tested the therapeutic effects of MSCs that were engineered to overexpress IL-10 when transplanted into rats following TBI in the medial frontal cortex.

**Methods:**

Thirty-six hours following TBI, rats were transplanted with MSCs and then assessed for 3 weeks on a battery of behavioral tests that measured motor and cognitive abilities. Histological evaluation was then done to measure the activation of the inflammatory response. Additionally, immunomodulatory effects were evaluated by immunohistochemistry and Western blot analyses.

**Results:**

A significant improvement in fine motor function was observed in rats that received transplants of MSCs engineered to overexpress IL-10 (MSCs + IL-10) or MSCs alone compared to TBI + vehicle-treated rats. Although tissue spared was unchanged, anti-inflammatory effects were revealed by a reduction in the number of glial fibrillary acidic protein cells and CD86 cells in both TBI + MSCs + IL-10 and TBI + MSC groups compared to TBI + vehicle rats. Microglial activation was significantly increased in the TBI + MSC group when compared to the sham + vehicle group. Western blot data suggested a reduction in tumor necrosis factor-alpha in the TBI + MSCs + IL-10 group compared to TBI + MSC group. Immunomodulatory effects were demonstrated by a shift from classical inflammation expression (CD86) to an alternative inflammation state (CD163) in both treatments with MSCs and MSCs + IL-10. Furthermore, co-labeling of both CD86 and CD163 was detected in the same cells, suggesting a temporal change in macrophage expression.

**Conclusions:**

Overall, our findings suggest that transplantation of MSCs that were engineered to overexpress IL-10 can improve functional outcomes by providing a beneficial perilesion environment. This improvement may be explained by the shifting of macrophage expression to a more pro-repair state, thereby providing a possible new therapy for treating TBI.

## Background

Traumatic brain injury (TBI) is the result of a sudden impact to the brain caused by things such as falls, car accidents, or a blast [1, 2]. One of the major causes of neurological damage and reduced functional outcome in TBI is due to the inflammatory response after injury. Following the initial injury, both pro- and anti-inflammatory cytokines are released, which initiate the infiltration of leukocytes and activate astrocytes and microglia to the injury site [3, 4]. The activation of such cells can be both beneficial and harmful to the outcome following TBI. Classical inflammation is known to release pro-inflammatory cytokines, such as interleukin-12, tumor necrosis factor-alpha (TNF-α), and interleukin-1β and express elevated levels of surface proteins, CD86, MHC-II, and CD80 [5]. Classical inflammation is also commonly associated with increased inflammatory responses, increased white matter injury, and cell death due to the release of pro-inflammatory cytokines that initiate the secondary injury cascade. Conversely, alternative inflammation is associated with reduction in inflammatory response and promotion of wound healing [6]. Alternative inflammation is typically known to release anti-inflammatory cytokines, with the signature cytokine released being IL-10 [7].

IL-10 is an anti-inflammatory cytokine that helps to regulate the immune response [8]. Previous work has shown that IL-10 can reduce inflammation after spinal cord injury, stroke, and TBI [9–11]. IL-10 is also capable of reducing astrogliosis after TBI due to the direct inhibition of the pro-inflammatory cytokine TNF-α [12, 13]. Furthermore, IL-10 is known to inhibit pro-inflammatory cytokines by reducing hemoglobin-induced oxidative tissue damage through the positive feedback loop with CD163, heme oxygenase-1, and IL-10 [14]. Moreover, the prolonged elevation of IL-10 following brain injury may be important for assisting in the resolution of the inflammatory response through the modulation of macrophage expression [8].

In addition to macrophages, IL-10 is released by other cell types, including mesenchymal stem cells (MSCs). MSCs have received considerable attention as a potential treatment for TBI [15, 16]. This is largely due to the ability to release pro-repair and anti-inflammatory proteins which reduce the inflammatory response [17]. However, MSCs consist of a very heterogeneous population, making it difficult to determine the extent to which subpopulations of MSCs release critical factors for reducing inflammation. However, since MSCs have a high capacity for undergoing genetic engineering, a viable option for delivering critical target proteins, such as IL-10, would be to insert the genes into MSCs and transplanting these altered cells into the damaged brain region. By directly transplanting these cells into the target brain region, it assures that the transplanted cells deliver the overexpressed gene and provide trophic support to the injured region.

By genetically engineering MSCs to overexpress IL-10, a stable infusion of IL-10 could be achieved following MSC transplantation into the brain. While systemic administration of IL-10 has previously been shown to be an effective tool to help ameliorate TBI symptoms by engineering MSCs to express IL-10, this could provide a synergistic effect that neither treatment could provide on their own [18]. Previously, this polytherapeutic approach, using MSCs and IL-10, has been shown to reduce inflammation in a collagen-induced model of arthritis [19], experimental autoimmune encephalomyelitis [20], ischemia-reperfusion injury in the lung [21], graft-versus-host disease [22], and ischemic stroke [11].

The purpose of the present study was to investigate if treatment with MSCs, as well as MSCs that have been engineered to overexpress IL-10, could reduce inflammation, exhibit pro-immunomodulatory effects, and improve functional recovery following contusion to the medial frontal cortex (MFC). To do this, measures of motor and cognitive abilities, quantification of preserved tissue, numbers of cells, microglia activation state, and protein levels of selected inflammatory markers were examined to assess the effectiveness of the transplanted MSCs and of the MSCs which were engineered to overexpress IL-10 in a contusion rat model of TBI.

## Methods

### Lentivirus construction

The IL-10 gene, from human complimentary DNA (cDNA), was amplified with forward primer 5′GCT AGC ATG CAC AGC TCA GCA CTG CTC 3′ and reverse primer 5′-GGA TCC TCA GTT TCG TAT CTT CAT TGT CAT GTA GG 3′. Restriction enzymes Nhe1 was added to the forward primer, and BamH1 was added to the reverse primer. The polymerase chain reaction (PCR) product was then cloned into the pGEM T-easy vector following the manufacturer’s protocol (Promega, Madison, WI). The plasmid was confirmed by PCR and by sequencing the cloning of IL-10 into the T-easy vector. IL-10 was digested with the restriction enzymes Nhe1 and BamH1 and ligated into the pLenti-CMV-SV40-GFP-2A-Puro vector (ABM Inc., New York, NY). Control plasmid pLenti-CMV-SV40-GFP-2A-Puro vector was made by self-ligating Nhe1 and BamH1. Plasmids pLenti-CMV-IL-10-SV40-GFP-2A-Puro and pLenti-CMV-SV40-GFP-2A-Puro were confirmed by PCR.

### Mesenchymal stem cells isolation

The bone marrow was extracted from the femur and tibia of an adult male, 3-month-old Sprague-Dawley rats. Following extraction, MSCs were grown in the following media: Alpha Modified Eagle’s Medium (Life Technologies, Carlsbad, CA) with 10% fetal bovine serum (Life Technologies, Carlsbad, CA) and 1% penicillin/streptomycin (Life Technologies, Carlsbad, CA). MSCs were selected by plastic cell adherence. Virus was produced in 293FT cell line by transient transfection. Lentivirus supernatant was collected and filtered through a 0.45-μm filter. At 70–80% confluency, the MSCs were infected with IL-10 lentivirus or control lentivirus in MSC media with 4 μg/mL polybrene (Sigma, St. Louis, MO). After 48 h of incubation, the media was changed, and puromycin was added (10 μg/mL; Sigma St. Louis, MO) to select for infected cells. Prior to transplantation, all MSCs were labeled with Hoechst 33258 (1:500; Life Technologies, Carlsbad, CA).

### Characterization of IL-10 engineered MSCs

#### Immunocytochemistry

Immunocytochemistry (ICC) was performed to confirm MSC surface markers and IL-10 expression. MSCs infected with IL-10 virus and control virus were grown on poly-l-lysine-coated (0.1% in H_2_0; Sigma, St. Louis, MO) 15-mm glass coverslips, until ~ 70–80% confluent. Cells were then fixed with 4% paraformaldehyde (PFA; Sigma, St. Louis, MO) for 10 min and washed 2 × 10 min in phosphate-buffered saline (PBS; 0.01 M at pH 7.4). Cells were then incubated in blocking buffer (10% Normal Goat Serum in 0.1% Triton X-100 in 0.01 M PBS) for 1 h at room temperature. To characterize MSCs, cells were stained with CD45 (1:200; Abcam, Cambridge, UK), CD90 (1:200; Abcam, Cambridge, UK), CD11b (1:250; Abcam, Cambridge, UK), CD105 (1:200; Abcam, Cambridge, UK), CD34 (1:100; Abcam, Cambridge, UK), and IL-10 (1:250; Abcam, Cambridge, UK). Primary antibodies were incubated overnight at 4 °C. Appropriate secondary antibody conjugated with florescent dyes was then applied for 1 h at room temperature. The cells were washed 3 × 10 min in 0.01 M phosphate buffer and mounted on slides with fluoromount media (Sigma, St. Louis, MO). Images for CD45, CD90, CD11b, CD105, and CD34 were taken using a fluorescent microscope (Zeiss, Oberkochen, Germany) at × 40, whereas the images for IL-10 ICC were taken using Leica fluorescent microscope at × 100 with oil immersion (Leica, Wetzlar, Germany). Additionally, to determine the expression of IL-10, the optical density (OD) of MSCs infected with IL-10 and control virus were measured using ImageJ software (NIH, Bethesda, MD).

#### RT-qPCR

To determine mRNA expression of IL-10 in the transduced MSCs, 3T75 flasks of MSCs, infected with the control virus (CMV-GFP), and 3T75 of MSCs, infected with the IL-10 virus (CMV-IL-10-GFP), were prepared for reverse transcriptase-quantitative polymerase chain reaction (RT-qPCR). First, cells were lysed by incubating them in 2 mL of Trizol (Life Technologies, Carlsbad, CA) for 5 min at room temperature. RNA was isolated from lysate according to RNeasy plus mini protocol (Quiagen, Hilden, Germany). Then, using the High-Capacity RNA-to-cDNA kit (Applied Biosystems, Foster City, CA), 1 μg of RNA was transcribed to cDNA. Six cDNA samples were tested for both MSC groups, and three replicates were run for each sample. RT-qPCR was then done using the Brilliant II SYBR Green protocol (Agilent Technologies, Santa Clara, CA). Expression levels of IL-10 (500 nM; forward primer 5′TGA TGC CCC AAG CTG AGA AC 3′; reverse primer 5′AAT CGA TGA CAG CGC CGT AG 3′) were measured, and glyceraldehyde 3-phosphate dehydrogenase (500 nM; forward primer 5′TCA ACA GCA ACT CCC ACT CTT CCA 3′; reverse primer 5′ACC CTG TTG CTG TAG CCG TAT TCA 3′) was used as a reference gene. Thermocycler settings were 10-min hot start at 95 °C, followed by 40 cycles starting at 95 °C, for 30 s, 52 °C for 1 min, and 72 °C for 30 s. Statistical analysis was performed using the 2^−ΔΔ*C*T^ method [23].

#### Western blot for cells

To determine protein levels of IL-10 in transduced MSCs, triplicate replicas of T75 flasks of either MSCs infected with the control virus (CMV-GFP) or MSCs infected with the IL-10 virus (CMV-IL-10-GFP) were used for Western blot analysis. Cells were grown to ~ 80% confluency, then the media was removed and the cells were washed and mechanically removed in tris buffer saline. Cells were then centrifuged, and the pellets were stored at − 80 °C. The cells were then lysed with radio-immuno-precipitation assay buffer [RIPA; 10 mM Tris-HCl (pH 8.0), 1 mM EDTA, 0.5 mM EGTA, 0.1% SDS, 140 mM NaCl, 0.1% sodium deoxycholate, 1% Triton X-100, with PMSF protease inhibitors (Sigma, St. Louis, MO)]. To obtain the supernatant, the homogenate was centrifuged at 20,000 g for 30 min at 4 °C. Protein concentrations of each sample were determined using the Pierce Bicinchoninic Acid Protein Assay (Thermo Scientific, Rockford, IL). To prepare samples to be run on a Tris-Glycine gel, equal parts of × 2 SDS-sample buffer [125 mM Tris-HCl (pH 6.8), 4% sodium dodecyl sulfate, 20% glycerol, 10% 2-mercaptoethanol, and 0.2% bromophenol blue] were added and then heated at ~ 95 °C for 2 min. Equal amounts of each sample were loaded into the well of the Tris-Glycine gel (4–20%; Invitrogen, Carlsbad, CA) and ran at 100 V for ~ 1 h in running buffer (25 mM Tris-Base, 192 mM glycine, 0.1% SDS). Proteins were then transferred to polyvinylidene difluoride membrane (Millipore, Billerica, MA) in transfer buffer (25 mM Tris–Base, 192 mM glycine, and 10% methanol) at 4 °C for overnight. Following transfer, membranes were washed 3 × 10 min in TBST and then blocked in 5% fat-free milk powder in TBST for 1 h. Primary antibodies were diluted in 5% fat-free milk in TBST as follows: rabbit anti-IL-10 (1:1000; Abcam, Cambridge, United Kingdom) and rabbit anti-β-tubulin (1:1000; Cell signaling, Danvers, MA) and applied to blots overnight at 4 °C. Membranes were washed, and appropriate secondary antibodies (1:10,000, goat anti-rabbit IgG; Santa Cruz, Biotechnology, Dallas, TX) diluted in 1.5% fat-free milk in TBST was applied for 1 h. Blots were developed with Immobilon Western Chemiluminescent horseradish peroxidase substrate (Millipore, Billerica, MA). Images were recorded using a gel documentation system (Bio-Rad Laboratories, Hercules, CA). OD of each band was measured using ImageJ software (NIH, Bethesda, MD).

### Rats

Thirty-nine male Sprague-Dawley rats (Charles River, Wilmington, MA), approximately 90 days old, were used in this study. Rats were pair-housed in a 12 h/12 h reverse light cycle, with lights turned on at 08:00 h, and with food and water provided ad libitum. Rats were randomly divided into four groups: TBI + vehicle (Hank’s balanced salt solution, HBSS; for vehicle; *n* = 10), TBI + MSCs (pLenti-CMV-GFP-2A-Puro; *n* = 10), TBI + MSCs + IL-10 (pLenti-CMV-IL-10-GFP-2A-Puro; *n* = 9), or sham + vehicle (*n* = 10). All procedures were approved by the Institutional Animal Care and Use Committee at Central Michigan University.

### Controlled cortical impact

Controlled cortical impact (CCI) model was performed according to procedures previously described by Peruzzaro and colleagues [24]. Rats were anesthetized using a mixture of 1–3% isoflurane (Henry Schein Co., Melville, NY) and 500-mL-1 L/min oxygen and maintained throughout surgery. Body temperatures were kept at 37 °C during surgeries, using a physiotemp machine (Physitemp Instruments, Clifton, NJ). Rats were placed in a stereotaxic instrument (Kopf Instruments, Tujunga, CA), and a midline incision was made to expose bregma. Sham rats receiving only an incision were then closed and allowed to recover. Rats receiving the CCI underwent a 6-mm craniotomy at 3-mm anterior to bregma (AP + 3.0, ML0.0). The impactor tip was placed over the exposed brain, and the cortex was compressed at a depth of − 2.5 mm at a velocity of 2.25 m/s with a duration of 0.5 s. Incisions were closed, and rats were allowed to recover. Animals were monitored 3 days, post-surgery for any signs of pain or discomfort.

### Stem cell transplantation

Transplantation surgeries were performed at 36 h after injury. The TBI rats were randomly divided into one of the three groups: MSCs + GFP, MSCs + IL-10-GFP, and HBSS. Sham animals were injected with HBSS. The rats were anesthetized and secured in the stereotaxic device as previously described for the CCI. Incisions from the CCI were then re-opened, and fascia was retracted, exposing the skull. A burr hole was drilled on either side of the skull, directly over the injection site. Injections were made bilaterally, at two depths in the surrounding area from the injury (AP + 3.0, ML ± 3.5, DV − 3.0, and − 1.5 mm from bregma). All rats received a 2-μL injection (100,000 cells/μl) at each site using a 10-μL Hamilton syringe at a rate of 0.33 μL/min. Overall, there were 4 injection points with a total of 8 μl, and therefore, a total of 800,000 cells injected per rat. The needle was left in place for 3 min after each injection and raised slowly to allow for appropriate diffusion. When performing the injections, the site at the deepest depth was completed first. The incisions were closed, and the rats were monitored until awake and for the following 3 days for any signs of pain and discomfort. After surgeries, MSC viability was confirmed with trypan blue.

### Behavioral assays

#### Morris water maze

A circular tank (134.62 cm in diameter and 57.15 cm high) was filled with water to a depth of ~ 26.67 cm and rendered opaque using non-toxic black tempera paint. A clear circular platform (13.97 cm in diameter and 24.13 cm high) was centered in the northeast quadrant of the tank at 2.54 cm under the water level for the 4 days of spatial ability testing. Cognitive flexibility was then tested by reversing the platform to the southwest quadrant of the tank for 3 subsequent days. Beginning on post-TBI day 7, the rats were placed at one of four start locations (North, South, East, or West) facing the wall [25, 26]. Location of the starting point was preselected by quasi-randomization of one short and one long distance away from the platform each day. Rats were given 90 s to find the platform. If rats were unable to find the platform, they were guided to it by hand. Once on the platform, rats were given 30 s to rest and observe the spatial cues before being placed in a holding cage for ~ 15 min. Rats underwent 2 trials per session with an inter-trial interval of 15 min, for 4 sessions in the Northeast quadrant followed by 3 sessions in the Southwest quadrant (post-TBI days 7–13), at approximately 16:00 h on testing days.

#### Ladder rung walking task

Ladder rung walking task is a motor test that assesses stepping and inter-limb coordination [27]. The apparatus consisted of a horizontal ladder, 99 cm long, with metal rungs each spaced 2.5 cm apart. The animal was placed at one end of the apparatus and encouraged to cross the ladder by presenting a food reward at the other end. Rats were video-recorded as they crossed the ladder. A trial was counted if the rat moved from one end of the apparatus to the other end without stopping. The number of foot faults (defined as when a paw moved below a ladder rung) was the primary dependent measure. The first and last five rungs were not counted in the recorded number of foot faults. Pre-training was performed for 2 days, with 3 trials per day. The last day of pre-training was scored for baseline, and 3 trials were conducted per day on post-TBI days 5, 12, and 19 at approximately 10:00 h on testing days.

#### Rotarod

Rats were tested on the accelerating rotarod to assess gross locomotor performance [24, 28]. The task consists of an elevated rotating rod (Columbus Instruments, Columbus, OH) that gradually accelerated from 6 to 30 RPMs over the course of 240 s. The rod was approximately 0.6 m from the padded floor of the apparatus and has a circumference of 24.13 cm. Rats were placed on the rod, in the direction opposite of the rotation. The latency was recorded until the rat either fell off the rod, the rat reached the maximum time of 240 s, or held on to the rod for greater than 1 rotation. Pre-operation training was performed for 4 days, 4 trials per day. On the last day, pre-training was recorded and the average of 4 trials was used as a baseline. Post-TBI testing was conducted with 4 trials per day with an inter-trial interval of 15 min on post-TBI days 18–22 at approximately 16:00 h on testing days.

### Histology

Histological analysis was done with the following number of rats in each group: TBI + vehicle = 7, TBI + MSCs = 7, TBI + MSCs + IL-10 = 6, and sham + vehicle = 7. Rats were euthanized with sodium pentobarbital (Fatal Plus, Vortech Parm, Dearborn, MI; 1 mL/4.54 kg) on post-TBI day 23. Rats designated for histology were transcardially perfused with 0.1 M PBS (Sigma, St. Louis, MO) followed by 4% PFA (Sigma, St. Louis, MO), to fix the tissue. The brains were removed and underwent further fixation in 4% PFA overnight at 4 °C and then transferred to 30% sucrose solution at 4 °C until the brains sank (~ 2–3 days). Next, the brains were frozen using 2-methylbutane packed in dry ice (Sigma, St. Louis, MO) and stored at − 80 °C until sectioning. The brains were then sectioned coronally at 30 μm on a cryostat (Vibratome UltraPro 5000, St. Louis, MO) set at − 20 °C. Tissue designated for hematoxylin and eosin (H&E) staining was directly mounted on positively charged slides (Thermo Scientific, Waltham, MA) and stored at − 20 °C. Immunohistochemistry selected tissue was stored in 0.01% sodium azide (Sigma, St. Louis, MO) in 0.01 M PBS at 4 °C.

#### Hematoxylin and eosin staining

Hematoxylin and eosin (H&E) staining was used to visualize the lesion. Brain slices at + 3, + 2, + 1, and 0 mm from bregma were stained to analyze preserved tissue. Tissue was warmed in an oven set at ~ 90 °C for 2 min to facilitate the adherence of the tissue onto slides. The H&E staining consisted of 1 min in tap water, 8 min in 0.1% hematoxylin (Meyers; Sigma, St. Louis, MO), 10 min under slowly running tap water, 30 s in 50% EtOH, and 2 min in 0.05% eosin (Sigma, St. Louis, MO). The tissue was then dehydrated by immersing slides in 50% EtOH, 70% EtOH, and 90% EtOH for 30 s each, and 100% EtOH for 1 min, after which the slides were cleared in xylene for approximately 1 min. Slides were cover slipped with Eukitt (Sigma, St. Louis, MO) and prepared for scanning. Images were scanned with Nikon Coolscan IV (Tokyo, Japan) and traced using ImageJ (NIH, Bethesda, MD) for assessing the amount of spared tissue. For sham rats, measurements were taken by tracing all the tissues. The mean area of the undamaged tissue was measured in the four brain sections and multiplied by 1 mm to account for the distance between each tissue section to find tissue volume.

#### Immunohistochemistry

Immunohistochemistry (IHC) designated tissue for fluorescent labeling first underwent an antigen retrieval step (1 M HCl, pH 0.09 [used for GFAP and Iba1]; Tris/EDTA, pH 9.0 [used for CD86 and CD163]) for 10 min. The tissue was washed in 0.01 M PBS for 3 × 10 min and then placed in blocking buffer (10% normal goat serum in 0.1% Triton X-100 in 0.01 M PBS) for 1 h at room temperature. The following primary antibodies were diluted in 0.1% Triton X-100 in 0.01 M PBS: glial fibrillary acidic protein (GFAP; for astrocytes; 1:5000; AVES, Tigard, OR) and ionized calcium-binding adapter molecule 1 (Iba1; for macrophage/microglia; 1:6000; Wako, Richmond, VA). The following primary antibodies were diluted in 0.3% Tween-20 in 0.01 M PBS: CD86 (for classical macrophage/microglia; 1:200; Bioss Woburn, MA) and CD163 (for alternative macrophage/microglia; 1:200; Hycult Biotech, Plymouth Meeting, PA). Primary antibodies were applied, and the tissues were incubated at 4 °C overnight. The tissue was washed, and appropriately conjugated secondary antibodies were applied (1:500, Alexa Fluor 488 goat anti-chicken Ig; 1:500, Alexa Fluor 594 goat anti-rabbit; 1:500, Alexa Fluor 594 goat anti-mouse, Invitrogen, Carlsbad, CA) for 1 h at room temperature.

Images were captured at × 20 objective using z-plane on a fluorescent microscope (Zeiss, Oberkochen, Germany). Cell counts were performed using 6 images around the lesion site in the frontal cortex (3 images from each hemisphere) and 2 images were taken at the CA1 and CA3 region of the hippocampus (1 image from each hemisphere). The mean number of cells was used for statistical analysis for cell counts using ImageJ (NIH, Bethesda, MD). To determine if there was a shift from classical to alternative macrophages/microglia activation state, the percentage from the ratio of CD163:CD86 was used to compare the TBI-injured groups. Microglial activation state was measured using a modified version of Colburn microglia response scale [29]. The Iba1 images taken in the frontal cortex were rated on a scale from 0 to 3. Scores were rated using the following criteria: 0 = resting microglia with long thin projection and cells well-spaced, 1 = mild response with projections still branched but less space between each cell, 2 = moderate response with projections less branched and cells occasionally overlapping, and 3 = intense response with short projections and cells increasingly overlapping.

### Western blot for tissue

To estimate levels of IL-10, TNF-α, and CD163, three rats in each group were designated for Western blot analyses. Animals were euthanized by rapid decapitation, and the brain tissue was immediately frozen in 2-methylbutane (Sigma, St. Louis, MO) and stored in − 80 °C until dissection. Each brain was dissected to isolate the cortex, which was homogenized in RIPA buffer. Western blots were conducted using the same protocol described above. Tris-Glycine gels used for Western blot cortical analyses, with 6% being used for CD163 and a 4–20% gradient being used for all others (Invitrogen, Carlsbad, CA). Primary antibodies were diluted in 5% fat-free milk in TBST as follows: rabbit anti-IL-10 (1:1000; Cell Signaling Technology, Danvers, MA), mouse anti-TNF-α (1:500; Novus Biological, Littleton, CO), mouse anti-CD163 (1:500; Hycult Biotech, Plymouth Meeting, PA), and rabbit anti- β-tubulin (1:1000; Cell Signaling Technology, Danvers, MA) and applied to blots overnight at 4 °C. Membranes were washed and appropriate secondary antibodies (1:10,000, goat anti-rabbit IgG; 1:5000, goat anti-mouse IgG, Santa Cruz, Biotechnology, Dallas, TX) were applied for 1 h. The OD of each band was measured with ImageJ and used for statistical analysis (NIH, Bethesda, MD).

### Statistical analysis

Measures of the ladder rung walking, tissue sparing, cell counts, and microglia response level scales were made by two blind experimenters and the means of each of those averaged for analyses. In all measures, the scores never deviated more than 10% between experimenters. All statistics were analyzed using SPSS with an *α* level of *p* < 0.05. Behavioral measures were analyzed using a repeated-measures analysis of variance (ANOVA). Greenhouse-Geisser correction was used when sphericity was not met. Tissue remaining, cell count, microglia response scale, and Western blots were analyzed using a one-way ANOVA. Tukey’s Honestly Significant Difference post hoc tests were conducted when appropriate. RT-qPCR was analyzed using a Student *t* test.

## Results

### Lentivirus cloning and construction

**Fig. 1:**
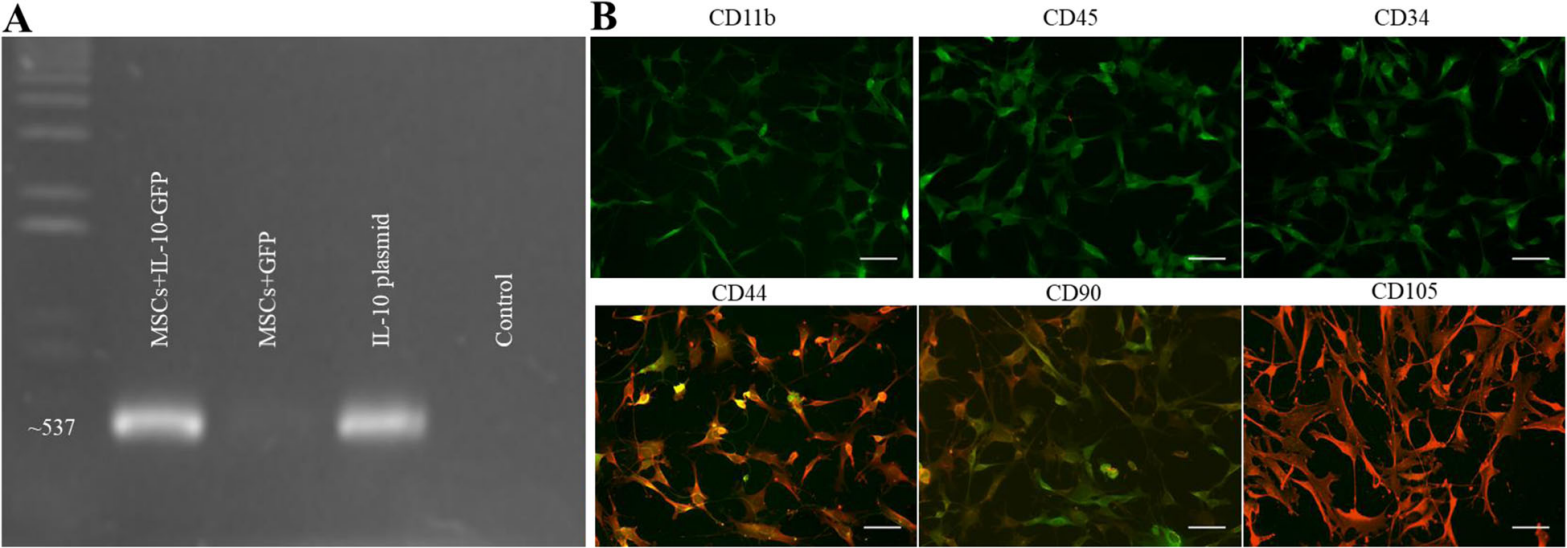
MSCs transduced with IL-10 lentivirus. **a** PCR product confirmed that IL-10 sequence was successfully integrated in the plasmid and detectable in transduced MSCs and absent in control virus plasmid and MSCs. **b** MSCs infected with lentivirus expressed GFP (green). MSCs were characterized by staining with the following antibodies: CD11b, CD45, CD34, CD44, CD90, and CD105 using appropriate secondary AlexaFlour 594 shown in the red. MSCs were negative for CD11b, CD45, and CD34. MSCs were positive for CD44, CD90, and CD105 (scale bar = 50 μm)

Cloning of human IL-10 into pLenti-CMV-GFP-2APuro was gene sequenced and confirmed that no mutation was present in the vector. PCR confirmed the presence of IL-10 with bands at ~ 537 base pairs in the pLenti-CMV-IL-10-GFP-2A-Puro and absent of IL-10 in the pLenti-CMV-GFP-2A-Puro (Fig. 1a).

### Characterization and transduction of MSCs

ICC revealed that both the IL-10 and control transduced MSCs were negative for CD11b, CD45, and CD34, and positive for CD44, CD90, and CD105 (Fig. 1b). MSCs infected with either IL-10 or control virus expressed GFP 2 days after transfection (Fig. 1b).

### Expression of IL-10 in MSCs

**Fig. 2:**
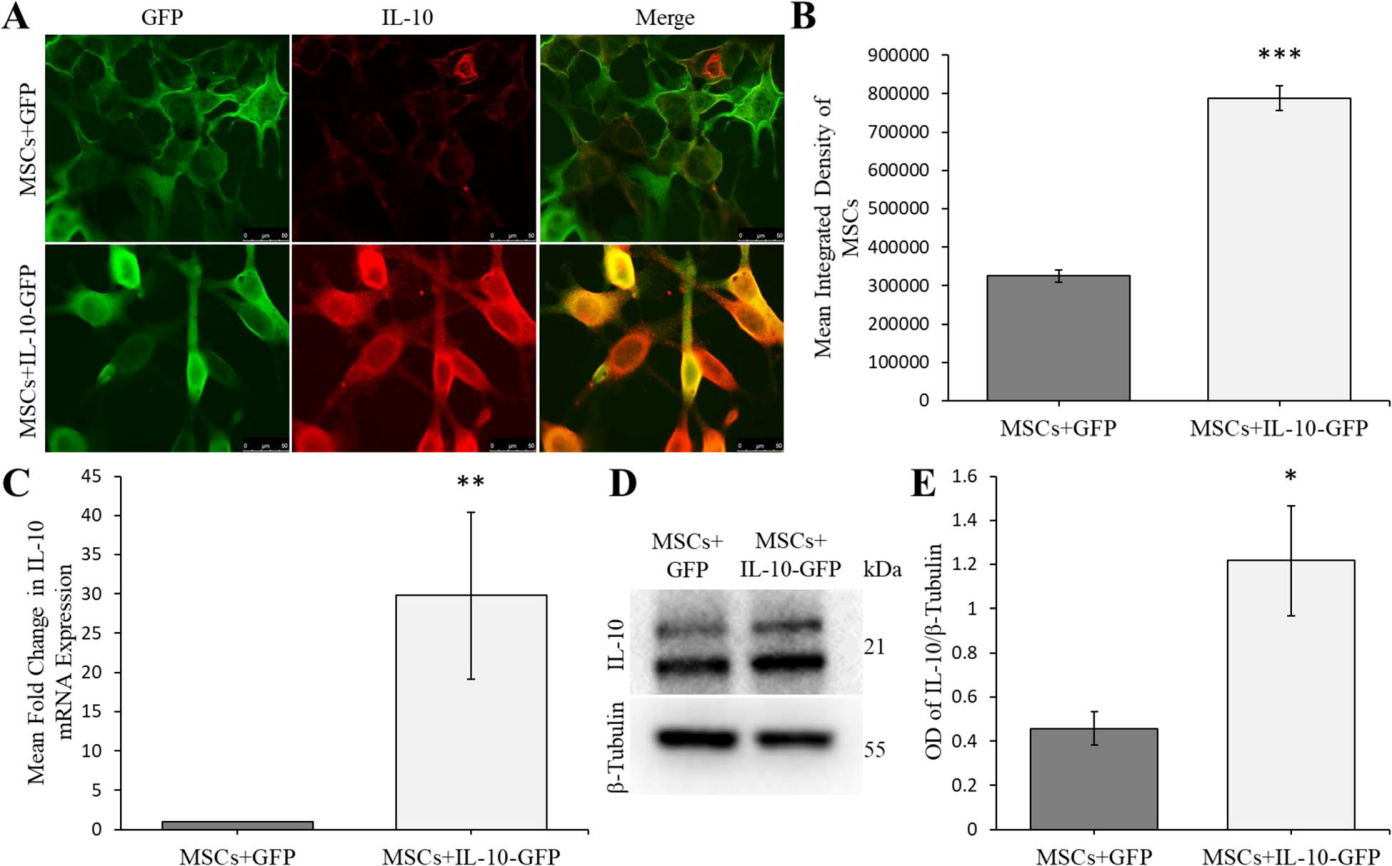
In vitro expression of IL-10 in transduced MSCs. **a** Representative immunocytochemistry images of MSCs with and without IL-10 overexpression. MSCs + IL10-GFP group appeared to have higher IL-10 immunofluorescent signal than MSCs + GFP group. **b** Mean integrated density of IL-10 showed that MSCs + IL-10 had significantly higher expression of IL-10 than MSCs + GFP (***p < 0.001). **c** RT-qPCR resulted in significant mean fold changes of IL-10 expression in MSCs + IL-10 group in comparison to MSCs + GFP (**p < 0.01). **d** Western blot IL-10 levels were compared between the two MSC groups. **e** MSCs + IL-10-GFP cells expressed a significantly higher amount of IL-10 level in comparison to MSCs + GFP (*p < 0.05; scale bar = 50 μm). Error bars represent standard error of the mean (± SEM)

A significant increase in IL-10 was observed, as indicated by an increase in mean-integrated density in MSCs infected with IL-10-GFP virus, compared to control MSCs (*t*(159.77) = 12.793, *p* < 0.001) (Fig. 2a, b). RT-qPCR revealed a significant mean fold change of IL-10, indicating that the IL-10 virus transduced MSCs beyond that of the control virus (*t*(17) = 3.188, *p* = 0.005) (Fig. 2c). Western blots performed on MSCs infected with IL-10 virus exhibited a significant increase in IL-10 protein, compared with MSCs infected with control virus (*t*(4) = 2.924, *p* = 0.043) (Fig. 2d, e).

### Behavior

#### Morris water maze

**Fig. 3:**
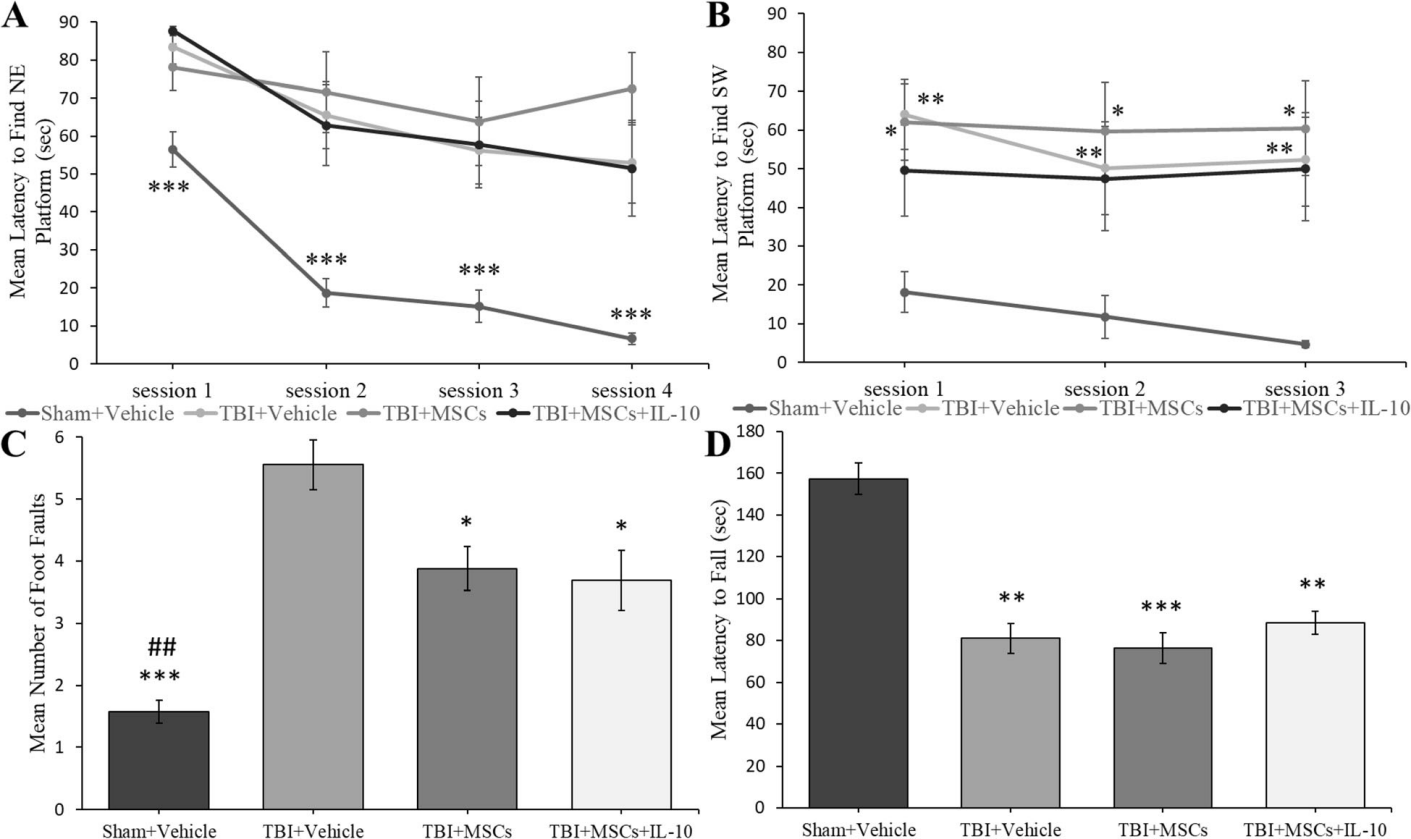
Behavioral outcomes after treatment with MSCs + IL-10. **a** Mean latency to find the platform in the MWM task across 4 days with platform in the northeast (NE) quadrant, all TBI groups took a significantly longer time to find the target platform than the sham + vehicle group (***p < 0.001). **b** Reversal trials with the platform in the southwest (SW) quadrant (mean latency across 3 days) indicated sham + vehicle group was significantly faster in finding the platform than TBI + vehicle (*p < 0.05) and TBI + MSCs (**p < 0.01) but not TBI + MSCs + IL-10. **c** Mean number of foot faults in the ladder rung walking task. Ladder rung test was run once a week for 3 weeks. TBI + vehicle group had significantly more foot faults than all other groups (*p < 0.05, ***p < 0.001), and the sham + vehicle group had significantly fewer foot faults than all other groups (##*p* < 0.01). **d** Mean latency (sec) to remain on the rotating rod in the rotarod test. The rotarod test was performed for 5 days, 4 trials per day. An injury effect was observed in TBI groups, whereas sham + vehicle group stayed on the rod significantly longer time than all other groups (**p < 0.01, ***p < 0.001). Error bars represent ± SEM

Mean latency (sec) to find the hidden platform (northeast platform target) in the Morris water maze task indicated that the performance for all rats significantly improved across days (*F*_2.423, 84.813_ = 19.889; *p* < 0.001). An injury effect was seen in all TBI groups as the sham + vehicle group had significantly shorter latencies to fine the platform than all groups (*F*_1, 35_ = 318.419; *p* < 0.001; Fig. 3a). During the reversal trials (platform in southwest quadrant), the sham + vehicle group found the platform significantly faster than TBI + vehicle (*p* = 0.014) and TBI + MSCs (*p* = 0.005; *F*_1, 35_ = 81.996, *p* < 0.001; Fig. 3b), but not the TBI + MSCs + IL-10 (*p* = 0.052).

#### Ladder rung walking task

No significant differences in foot faults were observed between groups at baseline (*p* > 0.05). Foot faults were diminished significantly within subjects across sessions (*F*_1.71, 59.863_ = 35.005; *p* < 0.001). The number of foot faults was significantly different between groups, post-TBI (*F*_1, 35_ = 276.863; *p* < 0.001; Fig. 3c). Tukey’s post hoc test revealed a reduced number of foot faults in the TBI + MSCs (*p* = 0.047) and TBI + MSCs + IL-10 (*p* = 0.028) compared to the TBI + vehicle group. However, neither treatment was capable of significantly returning function back to the sham + vehicle levels of the group (*p* = 0.004 and *p* = 0.01, respectively).

#### Rotarod

No significant differences in latency (sec) on rotarod were seen between groups at baseline (*p* > 0.05). Each group significantly improved rotarod performances across days (*p* < 0.001) during testing, and an injury effect was seen in all TBI groups compared to sham + vehicle group (*F*_1, 35_ = 211.78; *p* < 0.05; Fig. 3d), but no beneficial effects of the transplants were observed.

### Histology

#### Tissue sparing

**Fig. 4:**
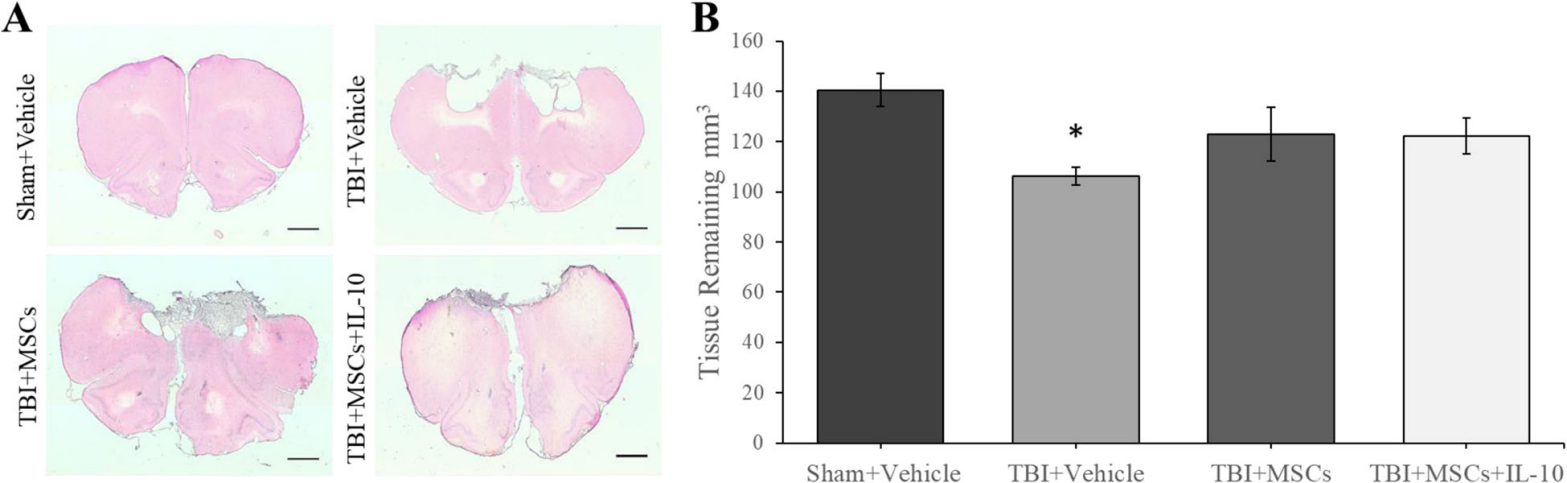
Mean volume of tissue spared were analyzed in rat brain sections at bregma + 3, + 2, + 1, and 0 mm. **a** Representative photomicrograph of lesions in the frontal cortex of each group. (Scale bar = 1 mm). **b** A significant reduction in tissue remaining was seen in TBI + vehicle group compared to sham + vehicle (*p < 0.05). Error bars represent ± SEM

A significant difference in tissue sparing was detected between groups (*F*_3,23_ = 3.629, *p* < 0.05; Fig. 4a, b). Tukey’s post hoc indicated that there was a significant sparing of tissue relative to the TBI + vehicle group in the sham + vehicle group (*p* = 0.015), but no significant differences were detected between TBI + MSCs + IL-10 (*p* = 0.355) and TBI + MSCs (*p* = 0.342) groups compared to sham + vehicle.

#### GFP expressing cells

Given that only sparse GFP and Hoechst labeling were detected near the needle track probably due to rejection of these cells, quantification of the number of transplanted cells could not be made with accuracy especially given the likelihood of autofluorescence [30].

#### GFAP

**Fig. 5:**
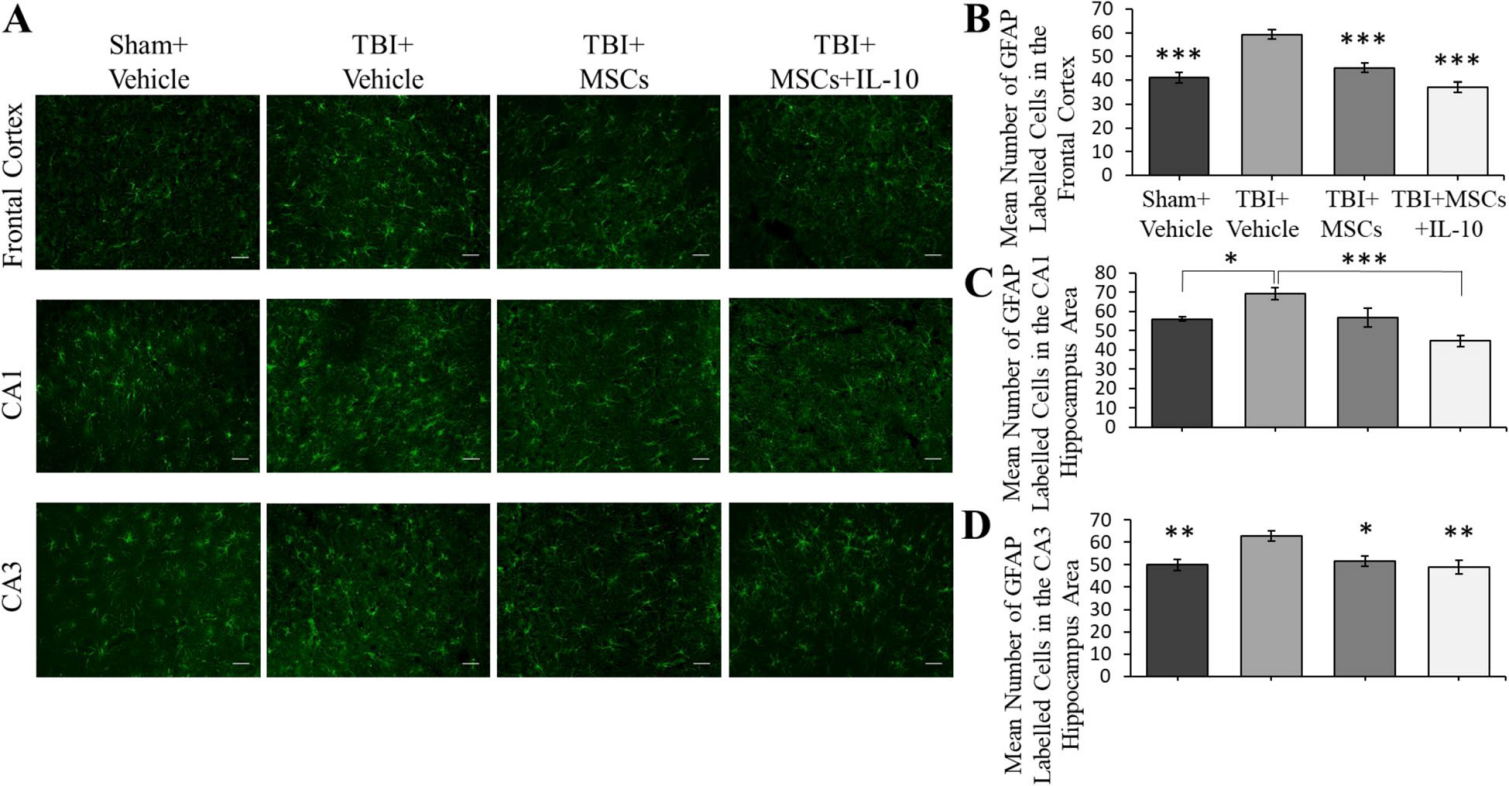
Astrocytes labeled with GFAP antibody in the frontal cortex and hippocampus. **a** Representive images of GFAP-postive cells in the frontal cortex and CA1 and CA3 region of the hippocampus. **b** A significant reduction in GFAP-positive cells was seen in TBI + MSCs (***p < 0.001) and TBI + MSCs + IL-10 (***p < 0.001) groups in the frontal cortex in comparison to TBI + vehicle group. **c** In the CA1 region of the hippocampus, a significant reduction of GFAP-positive cells were found in the TBI + MSCs + IL-10 (***p < 0.001) and sham + vehicle group (*p < 0.05) in comparison to TBI + vehicle group. **d** In the CA3 region of the hippocampus, TBI + vehicle group had a significant increase in GFAP-positive cells when compared to all other groups (*p < 0.05, **p < 0.01; scale bar = 50 μm). Error bars represent ± SEM

The number of GFAP-positive cells in the frontal cortex was significantly different between groups (*F*_3,23_ = 21.257, *p* < 0.05; Fig. 5). Tukey’s post hoc analysis revealed that the number of GFAP-positive cells was significantly increased in TBI + vehicle group compared to all other groups (sham + vehicle, TBI + MSCs, TBI + MSCs + IL-10; *p* < 0.001). A trend for fewer GFAP-expressing cells in the TBI + MSCs + IL-10 group, compared to the TBI + MSC group was observed (*p* = 0.057). The CA1 region exhibited a significant between-group differences (*F*_3,23_ = 8.459, *p* < 0.05). Tukey’s post hoc tests revealed that the treatment with MSCs + IL-10 significantly reduced the number of GFAP-positive cells compared to TBI + vehicle group (*p* = 0.001). The CA3 subfield of hippocampus also showed a significant group difference in the number of GFAP-positive cells (*F*_3,23_ = 6.412, *p* < 0.05; Fig. 5). Tukey’s post hoc tests indicated an increase in the number GFAP-expressing cells in TBI + vehicle group in comparison to sham + vehicle (*p* = 0.007), TBI + MSCs (*p* = 0.021), and TBI + MSCs + IL-10 (*p* = 0.005) groups.

#### Iba1

**Fig. 6:**
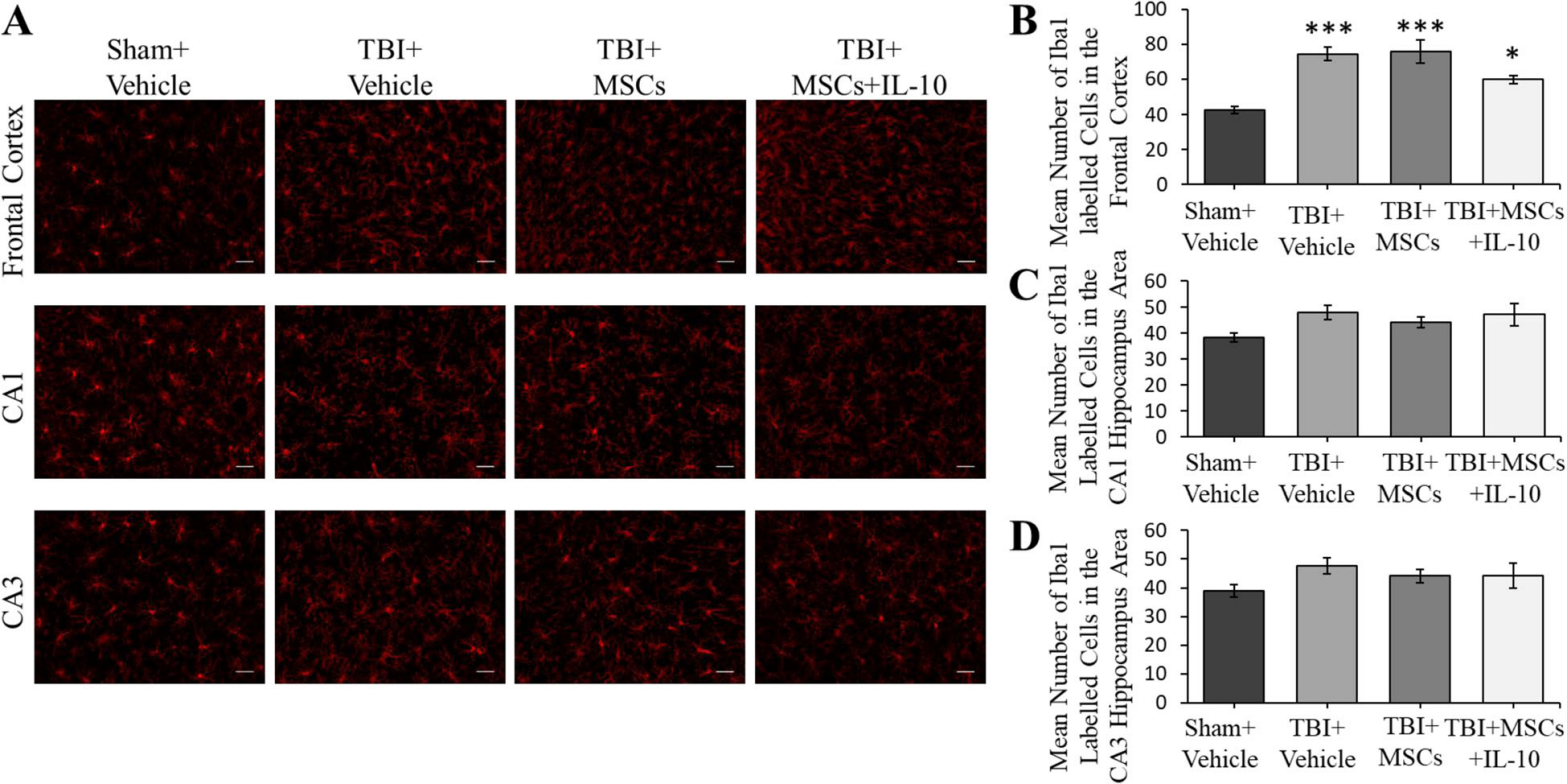
Macrophages/microglia labeled with Iba1 antibody in the frontal cortex and hippocampus. **a** Representive images of Iba1-postive cells in the frontal cortex and CA1 and CA3 region of the hippocampus. **b** In the frontal cortex, an injury effect was seen with an increased number of Iba1-positive cells in all TBI groups (*p < 0.05, ***p < 0.001). **c** No significant differences were seen among the groups in the CA1 region of the hippocampus. **d** No significant differences were seen among the groups in the CA3 region of the hippocampus (scale bar = 50 μm). Error bars represent ± SEM

Analysis of Iba1 showed a significant between-group difference in the number of Iba1-positive cells in the frontal cortex region (*F*_3,23_ = 14.074, *p* < 0.05; Fig. 6). Tukey’s post hoc test revealed that there was an increase in the number of Iba1-positive cells in all TBI groups relative to sham + vehicle group [(TBI + vehicle, (*p* = 0.001) TBI + MSCs, (*p* = 0.001) TBI + MSCs + IL-10 (*p* = 0.043)]. No significant between-group differences were seen in a number of Iba1-expressing cells in the CA1 (*F*_3,23_ = 2.531, *p* > 0.05) and CA3 (*F*_3,23_ = 1.527, *p* > 0.05) region of the hippocampus (Fig. 6).

#### Microglia activation state

**Fig. 7:**
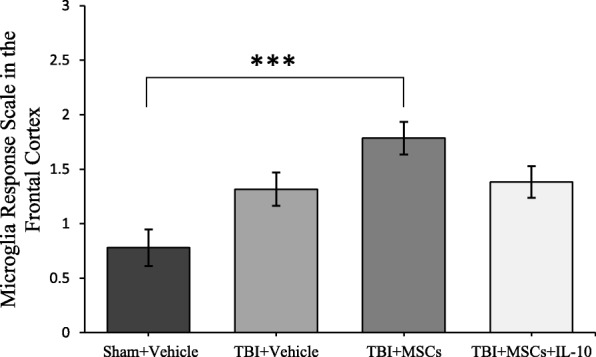
Microglia activation state in the frontal cortex. Microglia were more activated in TBI + MSC group compared to sham + vehicle group (***p < 0.001). Error bars represent ± SEM

The microglia activation state was significantly different between groups in the frontal cortex (*F*_3,23_ = 7.377, *p* < 0.05; Fig. 7). Tukey’s post hoc test revealed that there was an increase in activated microglia in the TBI + MSC group in comparison to the sham + vehicle group (*p* = 0.001).

#### CD163

**Fig. 8:**
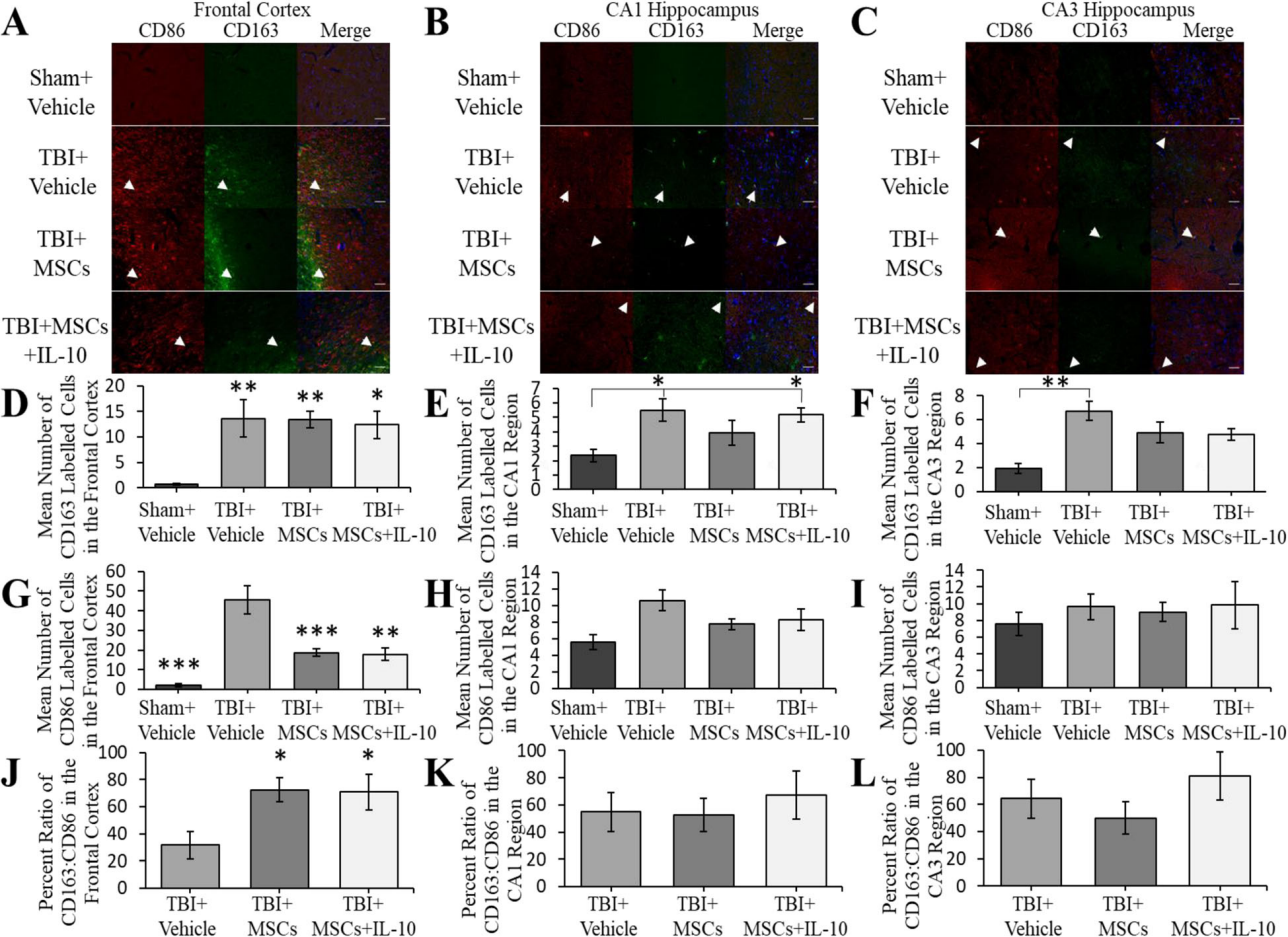
CD86 and CD163-labeled cells in the frontal cortex and hippocampus. **a**–**c** The images in the frontal cortex, CA1 region of the hippocampus, and CA3 region of the hippocampus are as follows: CD86 is in red, CD163 is green, and merge in yellow images show co-labeling of CD163 and CD86, arrows show an example of a co-labeled cell. Hoechst 33258 was used as a counterstain in blue (scale bar = 50 μm). **d** In the frontal cortex, a significant increase in the number of CD163-expressing cells was seen in all TBI groups compared to sham + vehicle group (*p < 0.05, **p < 0.01). **e** The mean number of CD163-labeled cells in the CA1 region of the hippocampus was significantly increased in the TBI + vehicle (*p < 0.05) and the TBI + MSCs + IL-10 (*p < 0.05) groups compared to sham + vehicle. **f** In the CA3 region of the hippocampus, CD163-positive cells were significantly increased in TBI + vehicle group compared to sham + vehicle (**p < 0.01). **g** Mean number of CD86-positive cells in the frontal cortex was significantly increased in TBI + vehicle group compared to all other groups (**p < 0.01, ***p < 0.001). **h**, **i** In the CA1 and CA3 region of the hippocampus, no significant differences in number of CD86-positive cells between groups were observed. **j**–**l** Cell counts for CD163 and CD86 were used to find the ratio of CD163:CD86 and multiplied by 100 to get the percent. **j** A significant shift to alternative macrophage/microglia state was seen in the frontal cortex (*p < 0.05). **k**–**l** No significant differences were observed among the groups in the CA1 and CA3 region of the hippocampus. Error bars represent ± SEM

The number of CD163-positive cells was significantly different between groups in the frontal cortex (*F*_3,23_ = 7.025, *p* < 0.05; Fig. 8). Tukey’s post hoc test revealed that there was an increase of CD163-labeled cells in all TBI groups (TBI + vehicle, *p* = 0.004; TBI + MSCs + IL-10, *p* = 0.013; and TBI + MSCs *p* = 0.005) relative to sham + vehicle. In the CA1 region of the hippocampus, significant differences were observed in the number of CD163-positive cells between groups (*F*_3,23_ = 4.299, *p* < 0.05; Fig. 8). Tukey’s post hoc exposed that TBI + vehicle (*p* = 0.017) and TBI + MSCs + IL-10 (*p* = 0.046) groups had significantly increased number of CD163-expressing cells when compared to the sham + vehicle group. In the CA3 hippocampal region, a significant difference in CD163-labeled cells was found between groups (*F*_3,23_ = 4.474, *p* < 0.05). Tukey’s post hoc showed a significant increase in the number of CD163-expressing cells in the TBI + vehicle group compared to sham vehicle group (*p* = 0.007).

#### CD86

The number of CD86-activated cells in the frontal cortex was significantly different between groups (*F*_3,23_ = 17.785, *p* < 0.05; Fig. 8). Tukey’s post hoc showed that TBI + vehicle group had significantly increased number of CD86-labeled cells relative to all other groups (TBI + MSCs, *p* = 0.001; TBI + MSCs + IL-10, *p* = 0.002; and sham + vehicle, *p* < 0.001). In the CA1 region of the hippocampus, a significant difference in the number of CD86-positive cells was observed (*F*_3,23_ = 4.221, *p* < 0.05). Tukey’s post hoc exhibited a significant increase in CD86-labeled cells in the TBI + vehicle group compared to sham + vehicle (*p* = 0.009). However, in the CA3 region of the hippocampus, no significant differences in the number of CD86-labeled cells were found (*F*_3,23_ = 0.340, *p* > 0.05; Fig. 8).

#### Co-labeling of CD86 and CD163

Co-labeling of CD86 and CD163 in the frontal cortex, CA1, and CA3 hippocampal region was observed in all TBI-injured groups (Fig. 8).

#### Macrophage/microglia shift

The percent ratio of CD163:CD86 was significantly different between groups in the frontal cortex (*F*_2,17_ = 4.835, *p* < 0.05; Fig. 8). Tukey’s post hoc showed a higher percentage of CD163 macrophages in the TBI + MSCs (*p* = 0.037) and TBI + MSCs + IL-10 (*p* = 0.044) groups than TBI + vehicle group. In the CA1 (*F*_2,17_ = 0.621, *p* > 0.05) and CA3 (*F*_2,17_ = 0.883, *p* > 0.05) region of the hippocampus, no significant differences were seen in the percent ratio of CD163:CD86 between all groups (Fig. 8).

### Western blot analyses

#### IL-10 levels

**Fig. 9:**
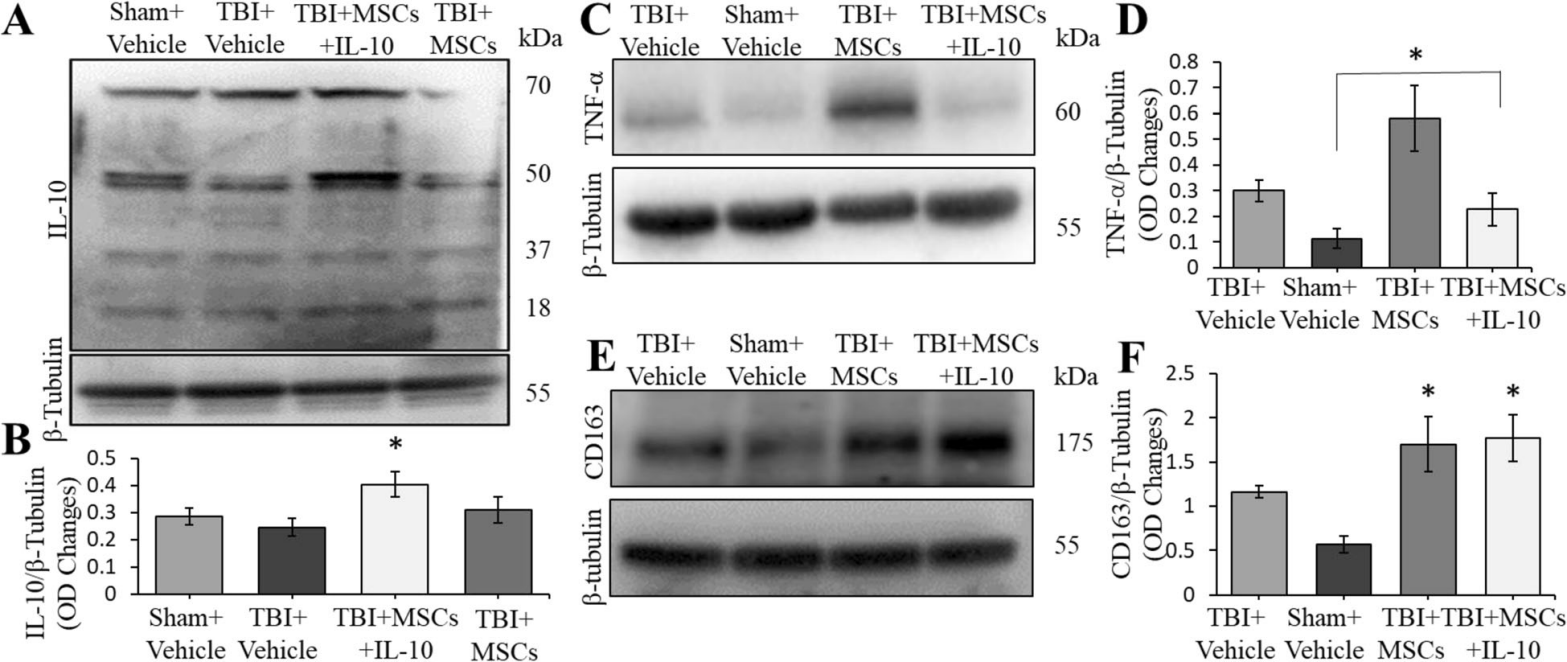
Western blots showed the levels of IL-10, TNF-α, and CD163 in the cortex. **a**–**b** IL-10 levels in TBI + MSCs + IL-10 group was elevated compared to TBI + vehicle (*p < 0.05). **c**, **d** TNF-α levels in the cortex was significantly elevated in TBI + MSC group when compared to all other groups (*p < 0.05). **e**, **f** CD163 levels were significantly elevated in TBI + MSCs and TBI + MSCs + IL-10 compared to sham + vehicle group (*p < 0.05). Error bars represent ± SEM

IL-10 levels in the cortex significantly differed between groups (*F*_3,8_ = 2.857, *p* < 0.05; Fig. 9a, b). Tukey’s post hoc analyses showed that IL-10 levels were significantly higher in TBI + MSCs + IL-10 group compared to TBI + vehicle (*p* = 0.042).

#### TNF-α levels

TNF-α cortical tissue was found to be significantly different between groups (*F*_3,8_ = 6.736, *p* < 0.05; Fig. 9c, d). Tukey’s post hoc analyses showed that there was an increase of TNF-α levels in TBI + MSC group relative to the TBI + MSCs + IL-10 (*p* = 0.047) and sham + vehicle (*p* = 0.011) groups.

#### CD163 levels

CD163 levels were significantly different between groups (*F*_3,8_ = 7.015, *p* < 0.05; Fig. 9e, f). Tukey’s post hoc analyses revealed that there was a significant increase in CD163 levels in both TBI + MSCs (*p* = 0.022) and TBI + MSCs + IL-10 (*p* = 0.016) when compared to sham + vehicle group. Additionally, no significant differences were seen between TBI + vehicle and sham + vehicle (*p* = 0.266) groups.

## Discussion

In this study, we demonstrated that MSCs were capable of being transduced to overexpress IL-10 in vitro and in vivo. In vivo treatment with MSCs overexpressing IL-10 or MSCs alone improved fine motor skills, prevented tissue loss, reduced number of activated astrocytes and macrophages, and shifted macrophage cell expression in the frontal cortex to a pro-repair state. Additionally, rats treated with MSCs engineered to overexpress IL-10 increased CD163 expression in the CA1, decreased GFAP-positive cells in the CA1, reduced microglial activation state, and decreased TNF-α in the cortex.

Severe MFC TBI, as in this model, creates a strong inflammatory response where production of inflammatory cytokines and recruitment of immune cells occur rapidly and can last for weeks after injury [18]. Prolonged inflammation after TBI is further suggested in our study by increasing the number of GFAP, Iba1, CD86, and CD163 labeled cells in the TBI + vehicle group. However, treatment with neither MSCs + IL-10, nor MSCs alone, was sufficient to reduce the number of Iba1- and CD86-positive cells. Although a significant sparing of cells was detected in the frontal cortex in the TBI groups, no beneficial effects of the transplants were demonstrated. This may be due to the fact that Iba1 labels both resting and activated macrophages and a more appropriate measure to detect macrophage activity may be to look at morphology of Iba1-labeled cells to determine the macrophage activity (i.e., resting or activated) [31]. This explanation is supported by our findings that the activation state of these microglia was significantly less in the MSCs + IL-10-treated group compared to the MSC-alone-treated group.

This study also demonstrated that treatment with MSCs + IL-10 or MSCs alone did not diminish the expression of CD163-positive cells. Multiple factors are known to upregulate CD163, such as glucocorticoids, IL-10, IL-6, and macrophage colony stimulating factor [32]. These factors are also known to be regulated by MSCs [33]. Furthermore, CD163-expressing cells are known to play a significant role in reducing the inflammatory response by creating a positive feedback loop with IL-10 and heme oxygenase-1 [14]. Additionally, CD163-positive cells are highly expressed on alternative M2 macrophages which are beneficial in phagocytosis of damaged cells and debris, which reduces the cytotoxic environment after TBI [32].

The IHC findings suggest that some macrophages expressed both CD86 and CD163, in both the frontal cortex and the hippocampus, were confirmed using the z-plane microscopic analyses. Although this represents the first observation of IHC expression of CD86 and CD163 co-labeling in TBI, it is supported by previous findings in which macrophages change their expressions over time and/or depending on the severity of injury [34]. Morganti and colleagues [35] found that after TBI, the distinction of typical M1 macrophage and M2 macrophage markers were not as clearly polarized as often described. IHC from the work of Morganti and colleagues [35] revealed co-labeling of CD36 (alternative inflammation marker) and MARCO (classical inflammation marker) as well as gene expression of both markers. Furthermore, Holladay and colleagues [34] demonstrated co-labeling of CD163 and CD80 (classical inflammation maker) in TBI. Although our paper looks at only a single marker typical for the classical and alternative phenotype, this work and others suggest a need to look at the temporal-spatial dynamics of each marker to better understand the functions of these macrophages/microglia.

MSC immunomodulatory effects occur because of release of multiple factors, such as IL-10. Therefore, the ratio of classical inflammation to alternative inflammation markers is an important index of functional recovery after injury [36]. These findings indicate that there was an upregulation of CD163 expressing cells, suggesting an increase in M2 phenotype macrophage/microglia in both groups treated with MSCs or MSCs + IL-10. This coincides with previous findings in which MSCs increased the recruitment of alternative macrophages to the injury site [37]. Our findings also suggest that treatment with MSCs, alone, were capable of shifting the ratio of alternative to classical macrophage/microglia phenotypes to an increased proportion of CD163 expressing cells around the injury site, at 3 weeks of post-CCI. Previously, Walker and colleagues [38] found that CCI-injured mice had similar changes in the ratio of alternative to classical macrophage phenotype at 120 h after treatment with adult progenitor cells.

Although anti-inflammation and pro-immunomodulatory effects were observed in our study, no MSCs were found 3 weeks after injury in both the MSCs + MSCs + IL-10 and the MSC alone groups. One possible explanation is that immune rejection of the MSCs although some studies suggest that MSCs can survive months after a TBI, the absence of GFP-fluorescing or Hoechst-labeled cells at 3 weeks post-CCI in the current study suggests that immune rejection of the cells occurred (Hasan et al., 2017). MSCs have previously been shown to be rejected within 3 weeks of transplantation by an increase in CD68-positive macrophages in the intact brain [39]. Although transplanted cell survival was not observed in the current study, the TBI + MSCs + IL-10 group still showed an increase in IL-10 production, as measured by Western blot (Fig. 9). Even though, IL-10 typically peaks early after injury (6–8 h) [8]. Furthermore, the increase in IL-10 in the TBI + MSC + IL-10 group may explain why no significant increase in TNF-α was seen at 3 weeks post-TBI, given that IL-10 is a direct inhibitor of TNF-α [40]. Additionally, a significant increase in TNF-α was detected in the TBI + MSC group, compared to sham + vehicle and TBI + MSCs + IL-10, suggesting a more pronounced inflammatory response in the TBI + MSC group.

This study also revealed mild improvements in functional outcomes with treatments of MSCs alone, as well as with MSCs + IL-10. These improvements included fine motor skills on the ladder rung walking test, but not on the rotarod test (Fig. 3). The ladder rung walking task is a sensitive test that shows abnormalities in fine motor skills, whereas the rotarod test primarily utilizes gross motor skills, which may explain why improvement was not observed in both of these tasks. However, Nakajima and colleagues [11] showed that rats which were treated intravenously with MSCs + IL-10 either immediately or at 3 h after a stroke had improved motor outcomes on the rotarod task. The discrepancy in these results may be due to the time and/or location in which the cells were transplanted or differences in the design of the testing parameters. Although no significant improvement was seen in the acquisition phase of the Morris water maze task, it is of interest to point out that the mean latency during the reversal sessions indicated that the TBI + MSCs + IL-10 rats were performing within the range of uninjured rats. Previous studies with either MSCs or IL-10 alone display conflicting results in their effectiveness at improving cognitive abilities [18, 41, 42].

## Conclusions

Collectively, these results suggest that MSCs engineered to overexpress IL-10 tended to reduce inflammation and promote functional outcomes to a greater extent than MSCs alone. Further investigations are needed to determine the optimal location and time point for transplanting these cells to improve their survivability and enhance functional outcomes. Given that both MSCs + IL-10 and MSCs are capable of modulating macrophage expression to increase the amount of CD163 expression, additional work may provide information on which trophic support or signaling molecules participate in the expression of CD163 to enhance this effect. As a proof-of-concept, this work provides additional support for the contention that transplantations of MSCs that are genetically altered to overexpress IL-10 can provide a possible new treatment that can modulate and reduce inflammation after TBI.

## Abbreviations

ANOVA: Analysis of variance
CCI: Controlled cortical impact
cDNA: Complimentary DNA
DAB: Designated for 3,3′-diaminobenzidine
EDTA: Ethylene-di-amino-tetra-acetic-acid
GFAP: Glial fibrillary acidic protein
H&E: Hematoxylin and eosin
HBSS: Hank’s balanced salt solution
Iba1: Ionized calcium-binding adaptor molecule 1
ICC: Immunocytochemistry
IHC: Immunohistochemistry
IL-10: Interleukin-10
MFC: Medial frontal cortex
MSCs: Mesenchymal stem cells
OD: Optical density
PBS: Phosphate-buffered saline
PCR: Polymerase chain reaction
PFA: Paraformaldehyde
RIPA: Radio-immuno-precipitation assay buffer
RT-qPCR: Reverse transcriptase-quantitative polymerase chain reaction
SDS: Sodium dodecyl sulfate
SEM: Standard error of the mean
TBI: Traumatic brain injury
TNF- α: Tumor necrosis factor-alpha
