# Correction to: Transplantation of mesenchymal stem cells genetically engineered to overexpress interleukin-10 promotes alternative inflammatory response in rat model of traumatic brain injury

**DOI:** 10.1186/s12974-021-02362-1

**Published:** 2022-01-11

**Authors:** S. T. Peruzzaro, M. M. M. Andrews, A. Al-Gharaibeh, O. Pupiec, M. Resk, D. Story, P. Maiti, J. Rossignol, G. L. Dunbar

**Affiliations:** 1 Field Neurosciences Institute Laboratory for Restorative Neurology, Central Michigan University, Mt. Pleasant, MI 48859 USA; 2 Program in Neuroscience, Central Michigan University, Mt. Pleasant, MI 48859 USA; 3 Department of Psychology, Central Michigan University, Mt. Pleasant, MI 48859 USA; 4 Field Neurosciences Institute, St. Mary’s of Michigan, Saginaw, MI 48604 USA; 5 Department of Biology, Saginaw Valley State University, Saginaw, MI 48610 USA; 6 Brain Research Laboratory, Saginaw Valley State University, Saginaw, MI 48610 USA; 7 College of Medicine, Central Michigan University, Mt. Pleasant, MI 48859 USA

## Correction to: Journal of Neuroinflammation (2019) 16:2 https://doi.org/10.1186/s12974-018-1383-2

The original version [1] of the article unfortunately contained mistakes in Figs. 5 and 6. The mistake occurred due to a copying and labeling error with image selection; all analyses remain the same.

It has been corrected in this correction (Figs. 5 and 6).

**Fig. 5:**
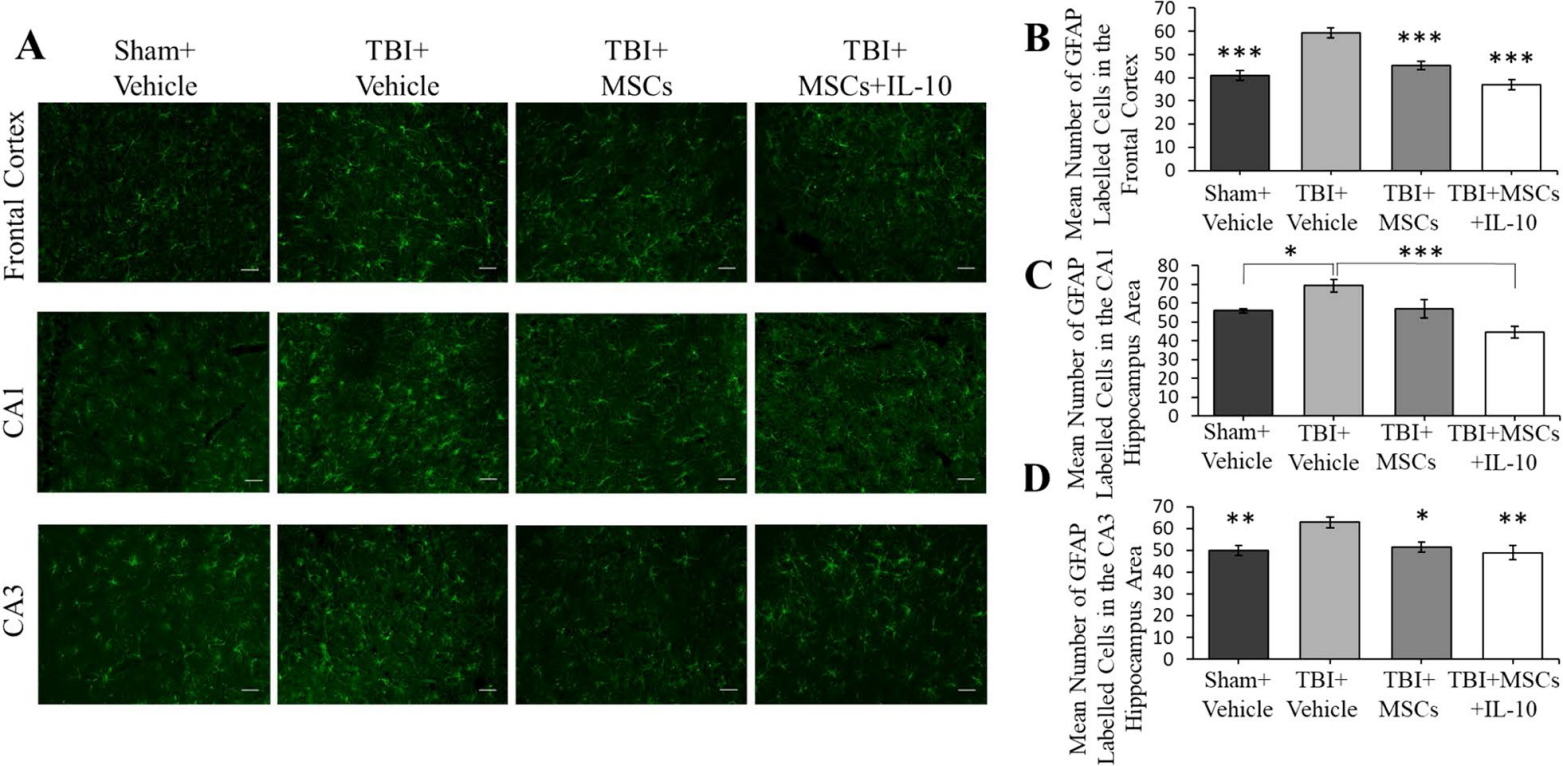
Astrocytes labelled with GFAP antibody in the frontal cortex and hippocampus. **A** A significant reduction in GFAP-positive cells was seen in TBI + MSCs (***p < 0.001) and TBI + MSCs + IL-10 (***p < 0.001) groups in the frontal cortex in comparison to TBI + vehicle group. **B** In the CA1 region of the hippocampus, a significant reduction of GFAP positive cells were found in the TBI + MSCs + IL-10 (***p < 0.001) and sham + vehicle group (*p < 0.05) in comparison to TBI + vehicle group. **C** In the CA3 region of the hippocampus, TBI + vehicle group had a significant increase in GFAP-positive cells when compared to all other groups (*p < 0.05, **p < 0.01; scale bar = 50 µm). Error bars represent ± SEM

**Fig. 6:**
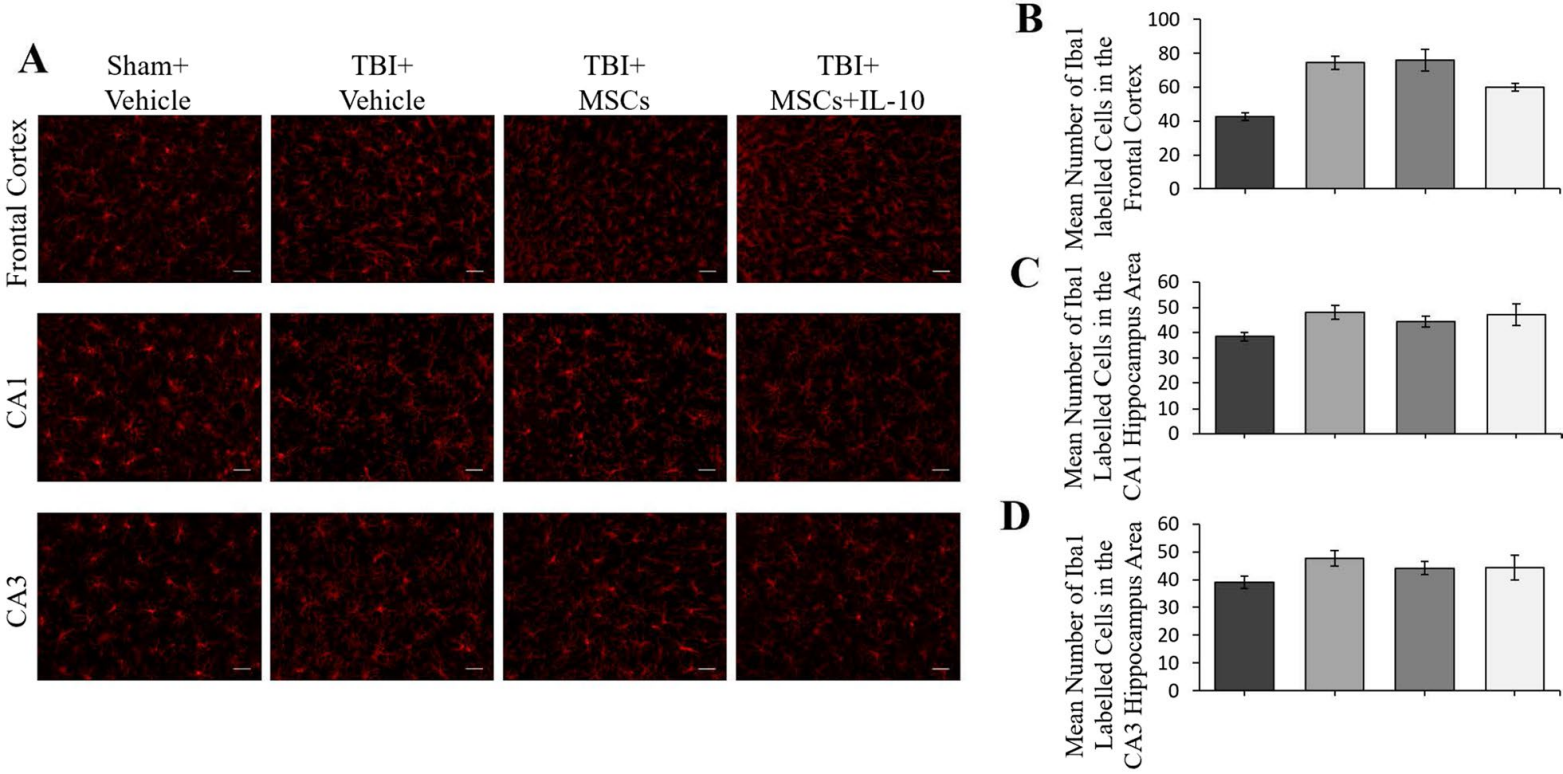
Macrophages/microglia labelled with Iba1 antibody in the frontal cortex and hippocampus. **A** In the frontal cortex an injury effect was seen with an increased number of Iba1 positive cells in all TBI groups (*p < 0.05, ***p < 0.001). **B** No significant differences were seen among the groups in the CA1 region of the hippocampus. **C** No significant differences were seen among the groups in the CA3 region of the hippocampus (scale bar = 50 µm). Error bars represent ± SEM
